# From pre-culture to solvent: current trends in *Clostridium acetobutylicum* cultivation

**DOI:** 10.1007/s00253-025-13428-y

**Published:** 2025-02-18

**Authors:** Katharina Oehlenschläger, Jan-Niklas Hengsbach, Marianne Volkmar, Roland Ulber

**Affiliations:** Department of Mechanical and Process Engineering, Institute of Bioprocess Engineering, RPTU Kaiserslautern-Landau, 67663 Kaiserslautern, Germany

**Keywords:** *Clostridium acetobutylicum*, ABE fermentation, Butanol, Pre-culture, ISPR, Co-cultivation, Renewable resources, Bioelectrochemical systems

## Abstract

**Abstract:**

The biological production of butanol via ABE (acetone-butanol-ethanol) fermentation using *Clostridium acetobutylicum* has a storied history of over 100 years, initially driven by the demand for synthetic rubber during World War I and later for industrial applications. Despite its decline due to the rise of petrochemical alternatives, renewed interest has emerged due to the global shift towards sustainable energy sources and rising oil prices. This review highlights the challenges in the cultivation process of *C. acetobutylicum*, such as strain degeneration, solvent toxicity, and substrate costs, and presents recent advancements aimed at overcoming these issues. Detailed documentation of the entire cultivation process including cell conservation, pre-culture, and main culture is seen as a fundamental step to facilitate further progress in research. Key strategies to improve production efficiency were identified as controlling pH to facilitate the metabolic shift from acidogenesis to solventogenesis, employing in situ product removal techniques, and advancing metabolic engineering for improved solvent tolerance of *C. acetobutylicum*. Furthermore, the use of renewable resources, particularly lignocellulosic biomass, positions ABE fermentation as a viable solution for sustainable solvent production. By focusing on innovative research avenues, including co-cultivation and bioelectrochemical systems, the potential for *C. acetobutylicum* to contribute significantly to a bio-based economy can be realized.

**Key points:**

*• Historical significance and revival of ABE fermentation with Clostridium acetobutylicum*

*• Current challenges and innovative solutions in cultivating C. acetobutylicum*

*• New avenues for enhancing productivity and sustainability*

## Introduction

**Fig. 1:**
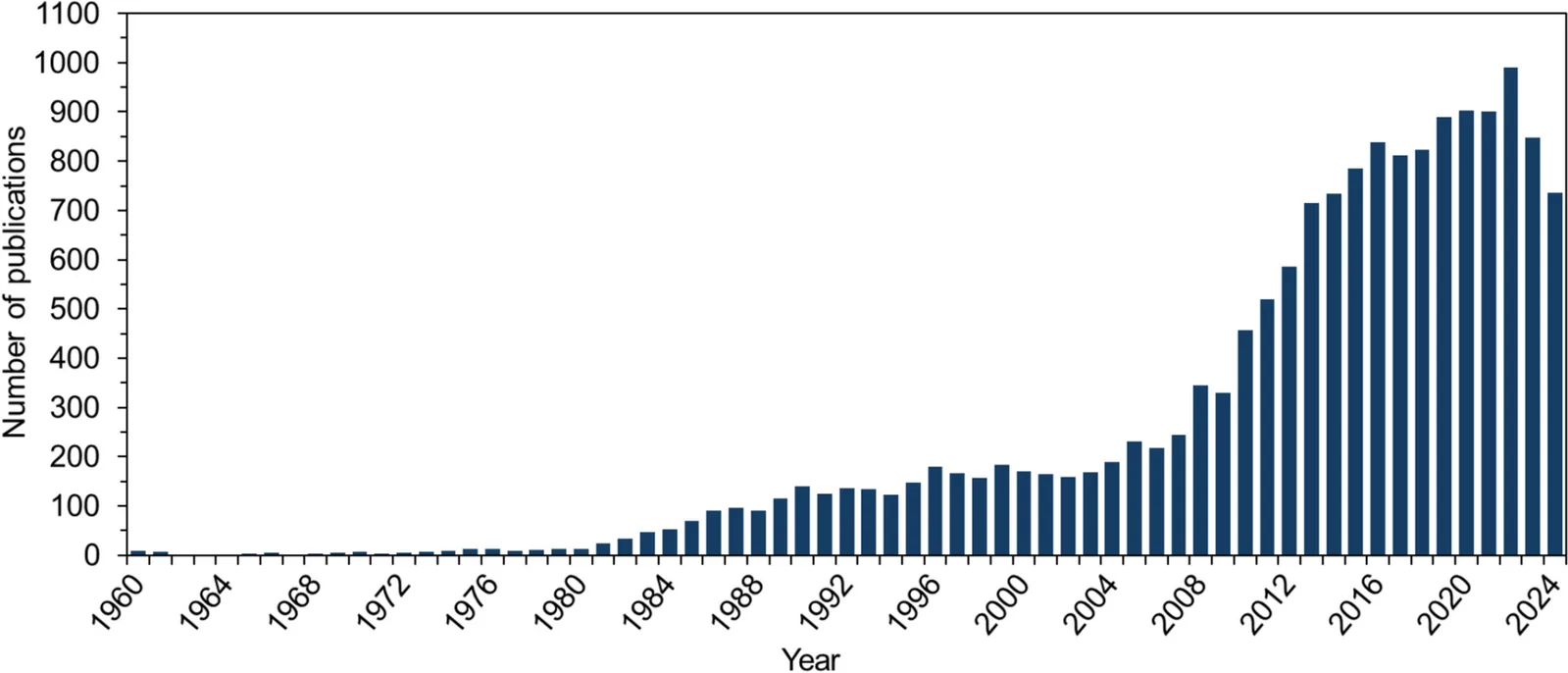
Number of publications related to *C. acetobutylicum* over the years (Scopus 2024)

The increasing demand for renewable energy sources and the necessity to reduce dependence on fossil fuels are driving increased interest in alternative production methods based on renewable resources. The biological production of butanol as part of ABE (acetone-butanol-ethanol) fermentation has a long and remarkable history starting in the early twentieth century due to the shortage of natural rubber and the demand for synthetic rubber (Gabriel and Crawford 1930). In this context, Chaim Weizmann isolated and cultivated the clostridium strain *Clostridium acetobutylicum* in 1914, which was capable of producing the solvents acetone, butanol, and ethanol (McNeil and Kristiansen 1986). The need for acetone in cordite production during both World Wars accelerated developments in ABE fermentation technology (Jones and Woods 1986). Further development of ABE fermentation was prompted by the automotive sector and its use of butanol as a paint solvent. In 1945 globally, 66% of all butanol was produced through the fermentation process (Dürre 2014). After the end of World War II, the ABE fermentation faced a considerable decline in application as the petrochemical industry continued to expand (Hasting 1971). The main limiting factors of the fermentation were the low product concentrations due to the toxicity of butanol and the associated high recovery costs. Today, growing concerns over the depletion of fossil resources and rising oil prices have led to a renewed focus on ABE fermentation with *C. acetobutylicum*. Significant research interest in *C. acetobutylicum* returned in 1980, followed by another notable rise around 2008 (Fig. 1).

This trend aligns with fluctuations in oil prices, indicating that rising oil costs, particularly during the 2008 financial crisis, have driven renewed scientific interest in *C. acetobutylicum* cultivation (Baumeister and Kilian 2016; Bhar and Malliaris 2011). Moreover, butanol is considered a potential biofuel due to its promising properties, including an energy density of 29.2 MJ·L^−1^ and lower hygroscopicity compared to ethanol (Dürre 2007). This growing interest in butanol has also led to an increased focus on *C. acetobutylicum* in recent years.

As interest in ABE fermentation resurges, understanding the metabolic processes of *C. acetobutylicum* becomes essential. The gram-positive, obligate anaerobic bacterium follows a biphasic metabolism consisting of acidogenesis and solventogenesis (Janssen et al. 2014). In the acidogenic phase, which aligns with the exponential growth phase, acids like butyrate and acetate are primarily produced. In the subsequent solventogenic phase, these acids are re-assimilated, and glucose is simultaneously converted into solvents such as acetone, butanol, and ethanol (Monot et al. 1982). It remains unclear what exactly triggers the transition from acidogenesis to solventogenesis. However, several factors are thought to influence this shift, including both intra- and extracellular concentrations of undissociated acids, particularly undissociated butyric acid (Terracciano and Kashket 1986; Maddox et al. 2000; Monot et al. 1984). Accordingly, internal and external pH also play a role in the metabolic shift (Haus et al. 2011; Bahl et al. 1982; Gottwald and Gottschalk 1985).

Despite decades of development, challenges such as the high cost of substrates, low yields, and toxic effects of the products on the bacteria still limit its large-scale production. Researchers today are once again focussing on basic cultivation conditions such as temperature and pH in order to achieve optimum process conditions (Feldmane et al. 2024). This reveals the significant research potential that is still present in this context. However, advances in optimizing the fermentation process, improving microbial strains, and use of alternative substrates make ABE fermentation with *C. acetobutylicum* a promising process for sustainable solvent production. This review provides an overview of current trends in *C. acetobutylicum* cultivation, focusing on the entire cultivation process including cell conservation, pre-culture, and main culture. A clearly defined cultivation process is essential to enable meaningful comparison and replication of data, which is an important step in advancing research towards the establishment of an economically viable process.

## Cultivation of *Clostridium acetobutylicum*

The cultivation of clostridia presents several challenges, including the metabolic shift from acidogenesis to solventogenesis, acid crash, and strain degeneration. To address these issues, it is crucial to have a well-defined cultivation method that covers all stages of the process, including cell conservation, pre-culture, and main culture. A standardized approach ensures consistent solvent production and helps maintain strain stability, ultimately improving the overall efficiency of the fermentation process.

### Cell preservation

Clostridial strain degeneration is a fundamental problem in industrial solvent production. It is caused by genetic changes that lead to a loss of the ability to produce solvents (Humphreys et al. 2022). This phenomenon is often triggered by repeated batch processing, subculturing, or continuous cultivation (Kashket and Cao 1995). A suitable cell preservation method is therefore of great importance. In the first half of the twentieth century, cells in industrial solvent production were typically preserved as spores in sterile sand, soil, or liquid cultures (Jones and Woods 1986; Awang et al. 1988). The spores were then heat-shocked at around 80 °C for a few minutes to induce germination. Sporulation in solventogenic clostridia is triggered by environmental stress such as nutrient starvation, high cell density, oxygen exposure, metabolite accumulation, or external pH (Diallo et al. 2021). Spores, being metabolically inactive but resistant to extreme environmental conditions including exposure to UV light, chemicals, heat, and oxygen, are particularly suitable for cell preservation. Today, cryopreservation has become a widely used technique that is also used in the preservation of clostridia cells. Cells are stored at temperatures below − 80 °C to slow down biochemical and metabolic reactions (Julca et al. 2012). Cryoprotectants such as glycerol, dimethyl sulfoxide, methanol, ethylene glycol, propylene glycol, and serum albumin are used to protect the cells against freezing damage (Hubálek 2003). Cell preservation in glycerol at low temperatures has been shown to recover high numbers of viable cells and maintain solvent production efficiency in subsequent cultivation of *C. acetobutylicum* (Gutierrez and Maddox 1987). The growth phase of microorganisms can play a crucial role in cell preservation, as it influences the viability and metabolic activity in subsequent cultivations. To ensure high survival ratios, cells harvested during the stationary growth phase are favored (Péter and Reichart 2001). In this context, it was also shown that *C. acetobutylicum* cells that were preserved from the stationary phase reached higher solvent concentrations in the subsequent cultivation compared to cells that were conserved in earlier stages of growth (Oehlenschläger et al. 2024). As the stationary growth phase is linked to solvent production and sporulation, the formation of spores may be responsible for the increased resistance to the freezing process. The connection between sporulation and solvent formation was first established by Jones et al. in 1982 through microscopic studies (Jones et al. 1982). Genetic studies in *C. acetobutylicum* confirmed that Spo0A acts as a multifunctional regulatory protein crucial for the transcription of both solvent formation and sporulation genes (Harris et al. 2002). In general, the cell preservation process of clostridia is not described in great detail in many publications. However, cell preservation is an essential element of the cultivation process, significantly contributing to the generation of high cell densities in pre-culture. Given the importance of cell preservation, it should be standard practice to include comprehensive documentation of preservation methods in scientific publications.

### Pre-culture

A standardized pre-cultivation procedure is crucial for achieving reproducible and comparable results in the main culture. Despite its importance, pre-culture conditions are rarely detailed in many publications. The cultivation conditions usually chosen are 35–37 °C at 150–200 rpm. Inoculation with 10 vol % pre-culture is also a standardized practice in most research studies involving clostridia. However, details about precise cell densities, incubation time of pre-culture, and the medium’s pH are often missing in both older and recent publications (Holt et al. 1984; Afschar et al. 1985; Capilla et al. 2022; Feldmane et al. 2024). In industrial ABE fermentation, achieving a sufficient inoculation volume required multiple build-up stages, with inoculation typically performed at 2–4 vol% (Jones and Woods 1986). Commonly used media for pre-cultivation are reinforced clostridia medium (RCM) and PY + X medium (Carl Roth GmbH + Co. KG 2024; Leibniz Institute DSMZ-German Collection of Microorganisms and Cell Cultures GmbH 2024). Both media consist of complex components including peptone and yeast extract, salt components, and 5 g·L^−1^ glucose. Alternatively, synthetic media without complex components, such as clostridial growth medium (CGM), are also used (Hartmanis and Gatenbeck 1984). The pH values of the media are specified by the manufacturer to be between 6.6 and 7. However, several authors use lower pH values of 5.8 and 6 for pre-cultivation (Hartmanis et al. 1986; Li et al. 2011). Investigations focusing on pH in pre-culture revealed that cell growth was observed only within the pH range of 5.0 to 6.2 when cryocultures served as the inoculum (Oehlenschläger et al. 2024). The incubation time of the pre-culture can also have an important influence, as it determines the growth phase of the bacteria. The growth phase of cells at the time of inoculation affects acid and solvent production in *C. acetobutylicum* main cultures. Inoculation with cells from the exponential growth phase leads to a short lag phase, rapid acid production, and low pH, resulting in an acid crash that prevents the metabolic shift (Sandoval-Espinola et al. 2015; Oehlenschläger et al. 2024). To solve this problem, a pH control has to be applied in the main culture (Gottwald and Gottschalk 1985). In contrast, using cells harvested from the stationary growth phase results in lower maximum growth rates and therefore lower acid production. This enables the shift to solventogenesis and ensures reliable solvent production in the main culture even without pH control (Oehlenschläger et al. 2024). Inoculation based on the cells’ growth phase, either exponential or stationary, is therefore more meaningful than relying on a specific time of incubation. Consequently, it is important to specify the growth phase of the cells, as has already been done in some publications (Bowles and Ellefson 1985; González-Pajuelo et al. 2005). The growth of the pre-culture can be monitored by measuring either cell density or pH. The exponential growth phase is marked by a drop in pH due to its association with acid production. In contrast, the transition to the stationary growth phase is linked to a slight rise in pH, driven by the re-assimilation of acids and the production of solvents. Monitoring pH thus allows for insight into the growth stage of cells and can act as a quality control parameter for the pre-culture. Defining pre-culture parameters such as medium, pH, and growth phase provides a basis for the comparability and reproducibility of different scientific studies which is a basic principle of scientific research.

### Main culture: from batch to continuous process

From the early to mid-twentieth century, ABE fermentation was already carried out on an industrial scale in various countries all around the world, including England, Canada, USA, Russia, Japan, South Africa, and China (Jones 2001). Fermentative solvent production was executed through a batch process, which was subsequently followed by distillation. Initially, the substrates, commonly corn mash or molasses, were sterilized in a pressure vessel, utilizing steam at temperatures reaching up to 130 °C (Gabriel 1928). Depending on the substrate, additional nutrients such as organic or inorganic nitrogen sources, phosphates, and buffering agents were supplemented to the medium (McNeil and Kristiansen 1986). The fermenters had a capacity of up to 900,000 L and were filled with 90 to 95% of the medium (Jones and Woods 1986). To create anaerobic conditions, the fermenters were flushed with carbon dioxide, which also served to mix the medium, as no mechanical stirrer was present in the vessels (McNeil and Kristiansen 1986). The fermentation process was conducted at temperatures between 29 and 37 °C with a cultivation duration of 40 to 50 h (McNeil and Kristiansen 1986). In batch processes, typically 18–22 g·L^−1^ solvents are produced with the Weizmann process yielding a characteristic product ratio of 6:3:1 for butanol, acetone, and ethanol (Jones and Woods 1986). Many aspects of this historical fermentation process for solvent production remain highly relevant to contemporary research. However, significant progress has been made in understanding the dynamics and control of solvent production in recent research.

The key to successful solvent production with *C. acetobutylicum* is to control the metabolic shift from acidogenesis to solventogenesis. During acidogenesis, the production of acids causes a decline in pH. In response, the transition to solventogenesis is triggered, wherein the acids are partially re-assimilated and pH-neutral solvents are produced. Acid production and the associated drop in pH can lead to an acid crash, which results in the loss of metabolic activity. The acid crash is usually associated with the concentration of undissociated acid, as these are able to diffuse across the cell membrane (Maddox et al. 2000; Cho et al. 2012; Monot et al. 1984). In the cell, the pH value is about 0.9 to 1.3 more alkaline and the acids dissociate into H + ions and anions, which results in a drop in the intracellular pH value (Gottwald and Gottschalk 1985). This reduces the proton gradient across the membrane and can lead to inhibition of nutrient transport (Cho et al. 2012). An established method to ensure the metabolic shift is therefore the use of a pH control, which prevents the pH from falling below a value of 4.5 (Monot et al. 1984). In this context, recent studies demonstrated that a pH above 4.4 is needed to keep the cells metabolically active and results in increased solvent production (Kumar et al. 2023). However, high pH values within the neutral range can hinder the metabolic shift and result in continued acid production as it is assumed that a specific amount of undissociated acid is required to trigger solventogenesis (Maddox et al. 2000). Therefore, pH regulation is an important factor to control the shift from acidogenesis to solventogenesis. In addition to the influence of pH, it is also known that the global regulator Spo0A and in particular the phosphorylation of the regulator play a crucial role as a trigger signal for the phase transition of *C. acetobutylicum* on protein level. Inactivation of the gene blocks the formation of the solvents. Recent results also indicate that the global regulator is additionally related to biofilm formation, which is typical for the solvent-forming phase of a *C. acetobutylicum* fermentation. Understanding this molecular relationship can be exploited in cultivation strategies, for example, to improve solvent tolerance and process robustness (Du et al. 2021; Yang et al. 2020). Media used for the cultivation of *C. acetobutylicum* are based on the work of Monot et al. from 1982, commonly referred to as P2-medium (Monot et al. 1982). This medium includes a carbon source like glucose, a nitrogen source, phosphate salts, vitamins, and minerals. Glucose concentrations around 50 g·L^−1^ are usually used to achieve maximum butanol concentrations in the batch process. Due to the high substrate costs, research in the twentieth century already focused on the use of alternative substrates including agricultural crops, cheese whey, apple pomace, algal biomass, bagasse, and rice straw (Jones and Woods 1986). Both cell growth and the efficiency of solvent production are significantly influenced by the medium composition, particularly the nitrogen source and mineral content. Consequently, supplementation of these media components is often required when alternative substrates are used (Bahl et al. 1986, Jin et al. 2024). Currently, there is a revival of interest in sustainable substrates particularly those derived from agricultural, agro-industrial, and municipal waste (Palaniswamy et al. 2023). The standard cultivation conditions, which have also been adopted from the past, are 35–37 °C and 150–200 rpm (McNeil and Kristiahsen 1985). These conditions have been further validated by recent research, where 37 °C and 200 rpm were defined as optimal cultivation conditions for *C. acetobutylicum* (Feldmane et al. 2024).

Within the batch process, the toxicity of butanol remains the primary limitation in ABE fermentation (Dürre 1998). As a result, product concentration cannot be increased through conventional fed-batch cultivation. However, there are approaches that enable fed-batch cultivation, utilizing immobilized cells by entrapment in calcium alginate. The hydrophilic properties of calcium alginate result in a lower solvent concentration within the gel matrix enabling butanol concentrations in the fermentation medium to reach up to 21.6 g·L^−1^ (Menchavez and Ha 2019). Nonetheless, the straightforward approach is to implement continuous cultivation. This requires cell retention or the immobilization of cells, which can be achieved through methods such as adsorption and entrapment of cells, or the use of carrier materials. The two-phase metabolism of solventogenic clostridia remains a challenge in continuous cultivation. Here too, pH control can be used to regulate metabolism. Studies on continuous solvent production with *C. acetobutylicum* showed that at high pH, around 5.7 acids are formed, while at a lower pH of 4.5, the process shifts towards solvent production (Haus et al. 2011; Bahl et al. 1982). Additionally, the use of a glucose and glycerol sugar mixture has been shown to be an effective method for controlling metabolism. As glycerol is more chemically reduced than glucose, this increases the production of reducing equivalents such as NADH, resulting in elevated NADH levels which support the production of solvents such as butanol (Vasconcelos et al. 1994; Andrade and Vasconcelos 2003). Another strategy for maintaining the cells in the solventogenic phase is the continuous addition of butyric acid aiming to inhibit the conversion of butyryl-CoA to butyric acid and to increase carbon flow towards butanol production (Chang et al. 2022; Huang et al. 2004). In this way, the highest productivity of 16.8 g·L^−1^·h^−1^ was achieved by Chang et al. using cells immobilized in a fibrous-bed bioreactor for continuous butanol production from glucose and butyrate (Chang et al. 2022). This process achieved a maximum butanol concentration of 9.5 g·L^−1^ and a yield of 0.24 g·g^−1^ considering both glucose and butyric acid as substrates. The authors attribute the high productivity and stability to the dense cell immobilization in the fibrous matrix that enabled the process to continue for over 1 month (Chang et al. 2022). Cell immobilization enhances the efficiency of the process by minimizing non-productive growth phases and enabling high cell densities, which improves product yield and volumetric productivity in bioreactors. Additionally, it protects cells from shear forces and provides stability against various environmental stresses (Zhu 2011). Materials such as wood pulp fibers, which are recyclable and biodegradable, can also be used as alternatives for immobilization (Survase et al. 2011).

Notably, many current investigations on the cultivation of *C. acetobutylicum* are revisiting foundational concepts and methodologies established in earlier decades. This continuity highlights the ongoing relevance of past research, especially as scientists seek sustainable solutions to modern challenges in biofuel production and waste management (Manasvi Vashisht et al. 2024; Riaz et al. 2022).

### In situ product removal

Despite the promising potential of ABE fermentation with *C.* *acetobutylicum*, the process has some inherent limitations that restrict its commercial utilization. In particular, the cytotoxic effect of the solvents produced poses a significant challenge as it leads to metabolic inhibition. This inhibition manifests itself in reduced substrate consumption and a decrease in overall cell metabolism with increasing product concentrations. Both fed-batch and batch fermentation processes reach their performance limits due to this problem (Bowles and Ellefson 1985; Fond et al. 1984). To address these challenges in bioprocessing, various in situ product removal (ISPR) approaches have been investigated in detail to increase the efficiency of solvent production with *C.* *acetobutylicum*. Different processes have emerged that can be integrated into the fermentation process for butanol or ABE recovery. This includes processes based on extraction, adsorption, pervaporation, and gas stripping methods, which have already been described and summarized in detail by Moon et al. (2016) and Xue et al. (2017)

In recent years, these processes have been further optimized and extensively studied. For the first time, a hybrid in situ method combining extraction and gas stripping was implemented in a 5-L pilot-scale packed-bed bioreactor to assess the scale-up potential of in situ recovery processes. During continuous ABE fermentation in this reactor, a high butanol productivity of 2.5 g·L^−1^·h^−1^ was achieved at a dilution rate of 0.5 h^−1^ (Chen et al. 2023). Additionally, a process utilizing Optipore L-493 resin for the adsorption of butanol, in which *C. acetobutylicum* NCIM 2337 was fermented on lignocellulosic hydrolysate derived from pineapple leaves, demonstrated significant improvement in ABE fermentation through the ISPR. This approach resulted in a 2.3-fold increase in yield (Sajjanshetty et al. 2021). Similarly, Tippkötter et al. introduced solvent-impregnated resins enclosing oleyl alcohol as an extractant that enabled the removal and recovery of butanol (Tippkötter and Roth 2020). Beyond this, more specialized adsorption materials were investigated for use in *C. acetobutylicum* fermentations. For instance, Vincent et al. evaluated poly(vinyldodecylimidazolium bromide) in a two-phase fermentation system, analyzing not only the biocompatibility but also the selectivity and partition coefficient for the ABE products. The results showed a high partition coefficient for n-butanol of 6.5 and a high selectivity (αBuOH/water = 46), demonstrating the material’s suitability for butanol recovery. This led to a 31% increase in volumetric productivity of the ABE fermentation compared to a control fermentation (Vincent et al. 2020). In another study, the integration of a continuous fermentation system consisting of four fixed bed biofilm reactors connected in series and an inline adsorption column resulted in an average butanol concentration of 24 g·L^−1^ and a productivity of 13 g·L^−1^·h^−1^ during fermentation with *C. acetobutylicum*. A glass column with the wet adsorbent Amberlite XAD-7 was used as the column for adsorption (Raganati et al. 2022). These new research results illustrate that the ISPR of ABE products in *C. acetobutylicum* fermentation is a key pillar for industrial application. However, none of them has yet been optimized to the point where they can be implemented on an industrial scale, although numerous promising techniques have already been developed.

## Current trends for sustainability and improved solvent production

Based on the fundamental cultivation process, new innovative cultivation methods and strain engineering strategies are gaining increasing attention. These methods offer promising approaches to improving efficiency and sustainability in solvent production with *C. acetobutylicum*. Especially, the use of lignocellulosic biomass as a valuable feedstock for biobutanol fermentation not only addresses the issue of waste but also plays a crucial role in advancing sustainable practices and a circular bioeconomy.

### Fermentation on renewable resources

The use of alternative substrates, such as starchy but also lignocellulosic raw materials, in ABE fermentation, is not a recent development, as various feedstocks were already explored in the past century (Jones and Woods 1986). The focus on alternative substrates for clostridia fermentations was largely driven by economic constraints and resource shortages, especially during wartime. Substrates like molasses, a side stream from sugar production, were already being used for ABE fermentation. These efforts primarily aimed to leverage readily available and inexpensive by-products of existing industries. Modern research emphasizes sustainability, resource efficiency, and the principles of the circular economy for substrate selection. In contrast to a linear economy, a circular economy strives to close material loops, meaning that all occurring material streams are considered resources (Nobre and Tavares 2021). This also applies for material which is currently considered waste. Many side or waste streams of current production processes consist of lignocellulosic biomass, which is a typical example of a renewable resource. Renewable resources are organic resources from agriculture, forestry, or fishing which are no part of the food or feed industry and are used energetically or materially. Lignocellulosic biomass consists of the three main components lignin, hemicellulose, and cellulose in different proportions, depending on the source of the material (Joshi and Manjare 2024). While lignin is a heteropolymer of phenols, hemicellulose consists of pentoses and hexoses, and cellulose is a polymer of glucose molecules. As the structure of lignocellulose is very recalcitrant, a pretreatment of the biomass is imperative to obtain the carbohydrates contained. Pretreatment methods can be classified according to the underlying principles, such as physical, chemical, physicochemical, or biological pretreatment processes (Langsdorf et al. 2021). Usually, several methods are combined for a successful pretreatment of the biomass. There are numerous comprehensive reviews presenting different pretreatment methods (Joshi and Manjare 2024; Abolore et al. 2024; Langsdorf et al. 2021; Amiri and Karimi 2018). Prevalent methods employ acids, alkalis, or solvents. After breaking up the recalcitrant lignin structure, the residue is usually saccharified enzymatically. Hereby, saccharolytic enzymes degrade hemicellulose and cellulose into monosaccharides which can be metabolized more easily by the fermenting microorganism.

Nowadays, further side streams from production processes which are currently not valorized yet are explored as a potential feedstock. Table 1 gives an overview of recent publications which use lignocellulosic biomass from different origins as feedstock for fermentations with *C. acetobutylicum*. *C. acetobutylicum* has demonstrated the ability to efficiently metabolize a range of sugars derived from lignocellulosic biomass, including glucose, mannose, galactose, arabinose, and xylose (Raganati et al. 2015; Survase et al. 2011). The strain preferentially utilizes glucose over pentoses like xylose or arabinose, a phenomenon known as carbon catabolite repression (CCR), where the presence of glucose inhibits the utilization of pentoses (Ounine et al. 1985). However, genetic engineering approaches aim to enable the simultaneous fermentation of pentoses and hexoses by inactivating genes that encode transcriptional repressors (Delarouzée et al. 2023). A very common example of lignocellulosic biomass valorization is the use of side streams from agriculture, mostly parts of plants which are not used as food such as sugarcane bagasse and straw of different grains (Reis Kemita et al. 2024; Luo et al. 2024; Liu et al. 2024; Muniasamy et al. 2024). In the food industry, a lot of side streams are produced as well, which are hitherto considered waste. This also touches on the problem of food waste in general. These wastes can be valorized as feedstock for the fermentative production of biobutanol (Jin et al. 2024; Avcı et al. 2023; Tigunova et al. 2023; Suresh et al. 2024). Avcı et al. demonstrate the suitability not only of fresh potatoes as feedstock for the production of biobutanol with *C. acetobutylicum*, but also of rotten potatoes, which have currently no other application. Final butanol and ABE concentration and the yields from rotten potatoes are very similar to the results of fresh potatoes (Avcı et al. 2023). Jin et al. used an enzyme complex to reduce the viscosity of sweet potato mash, leading to a 10% increase in butanol concentration (Jin et al. 2024). Also, apple pomace from the juice production (Tigunova et al. 2023) and spent mushroom residues were successfully used as substrates (Suresh et al. 2024). As the material was already processed for food production, usually no or only mild further pretreatment is necessary prior to the use in fermentations, which makes side streams from the food industry especially interesting. The same applies for the use of agave juice, an unused material stream from the fiber production (Oliva-Rodríguez et al. 2024). Due to the high lignin content of woody biomass, harsher pretreatment is necessary to make it accessible for microbial processing (Dou et al. 2021, 2018; Tippkötter et al. 2014a). Traditional municipal waste, which needs to be disposed of by the city administration, was also shown as suitable feedstock (Volkmar et al. 2023; Farmanbordar et al. 2024). The wide range of lignocellulosic side streams presented here demonstrates not only the flexibility of the biotechnological processes for ABE fermentation but also underlines the creative possibilities for implementing a circular bioeconomy. Innovative pretreatment methods for lignocellulose, like the use of a ternary deep eutectic solvent consisting of choline chloride, succinic acid, and glycerol, effectively improve the disruption of the lignocellulose structure. This pretreatment enhances glucan digestibility, leading to almost complete hydrolysis yields and higher sugar release (Luo et al. 2024). Also, the combination of alternative pretreatment methods, including microwave radiation, ultrasonication, and alkali treatment, improves biomass digestibility and leads to a higher sugar release from lignocellulosic biomass (Suresh et al. 2024). The detoxification of lignocellulosic hydrolysate is another new approach to improve fermentation performance. As a detoxification method, Liu et al. used a strong acid cation exchange resin to remove furfural and 5-hydroxymethyl furfural from hydrolysate. This approach led to a sixfold increase in butanol and solvent concentrations compared to the use of untreated hydrolysate (Liu et al. 2024). In addition to lignocellulosic biomass, other waste streams, such as those from palm oil or biodiesel production that are rich in glycerol, have also been used for solvent synthesis with clostridia (Johnson and Rehmann 2016; Tippkötter et al. 2014b). By expanding the array of feedstocks used, the reliance on conventional resources decreases, fostering a more resilient and sustainable production framework while minimalizing waste at the same time. The integration of alternative feedstocks, coupled with advanced pretreatment and detoxification techniques, holds great promise for improving fermentation efficiency, fostering sustainability, and advancing the principles of a circular bioeconomy. However, the challenges in the utilization of alternative feedstock include not only the accessibility of sugar sources and the control of inhibitors but also the availability of nutrients. Therefore, when utilizing renewable raw materials, nutrients typically need to be supplemented (Liu et al. 2024, Tigunova et al. 2023; Jin et al. 2024). Furthermore, additional challenges such as economic feasibility and scalability of the process remain fundamental issues. Ongoing research in strain development and process optimization will be crucial in overcoming these challenges.

**Table 1:** Overview of recent publications using different renewable materials as feedstock for fermentation with *C. acetobutylicum* to produce butanol or ABE solvents

Material	Pretreatment	Cultivation	Butanol	Total solvent	Reference
Sugarcane bagasse	Dilute acid pretreatment, enzymatic saccharification	Batch	9.5 g·L^−1^	15.7 g·L^−1^ 0.36 g·g^−1^	Reis Kemita et al. (2024)
Corn stover	Ternary deep eutectic solvent + 1% H_2_SO_4_, enzymatic saccharification	Fed batch	11.9 g·L^−1^	20.7 g·L^−1^	Luo et al. (2024)
Wheat straw	Dilute acid pretreatment, enzymatic saccharification, detoxification with strong acid cation exchange resin 001 × 7	Batch	7.42 g·L^−1^	12.97 g·L^−1^	Liu et al. (2024)
Sorghum straw	Protic natural deep eutectic solvents, enzymatic saccharification	Fed batch, stirred tank bioreactor	0.36 g·L^−1^·h^−1^	17.5 g·L^−1^	Muniasamy et al. (2024)
Sweet potato processing waste	Enzymatic reduction of viscosity	Batch	11.39 g·L^−1^	18.28 g·L^−1^ 0.37 g·g^−1^	Jin et al. (2024)
Potatoes, fresh	Smashing, addition of water, autoclaving	Batch	11.4 g·L^−1^	17.6 g·L^−1^ 0.29 g·g^−1^	Avcı et al. (2023)
Potatoes, rotten	Smashing, addition of water, autoclaving	Batch	11.2 g·L^−1^	17.1 g·L^−1^ 0.32 g·g^−1^	Avcı et al. (2023)
Apple pomace after juice production	No further pretreatment	Batch	6.00 g·L^−1^	N/A	Tigunova et al. (2023)
Spent *Pleurotus ostreatus*	Hybrid treatment with alkali, microwave and ultrasonification, enzymatic saccharification	Batch	9.84 g·L^−1^ 0.38 g·g^−1^	10.8 g·L^−1^ 0.40 g·g^−1^	Suresh et al. (2024)
*Agave lechuguilla* juice	No further pretreatment	Batch, bioreactor	5.96 g·L^−1^	N/A	Oliva-Rodríguez et al. (2024)
Willow biomass	Acid-catalyzed steam explosion, enzymatic saccharification	Batch	8.5 g·L^−1^ 0.21 g·g^−1^	12.7 g·L^−1^ 0.31 g·g^−1^	Dou et al. (2021)
Municipal green waste	Organosolv pretreatment, enzymatic saccharification	Batch	N/A	0.31 g·g^−1^	Volkmar et al. (2023)
Municipal biowaste	Organosolv under mild conditions; enzymatic saccharification	Batch	N/A	0.11 g·g^−1^	Farmanbordar et al. (2024)

*N/A* not available

### *Clostridium acetobutylicum* in bioelectrochemical systems

In addition to the use of renewable raw materials, other fields of research focus on the increase of solvent production of *C. acetobutylicum*. One important research focus is electrobiotechnology, in which targeted attempts are made to channel the metabolism towards more highly reduced end products such as butanol by adding external electrons. This involves the use of bioelectrochemical systems (BES), which are often equipped with a three-electrode system to apply a specific electric potential.

BES with whole-cell catalysts, such as *C. acetobutylicum*, *C. pasteurianum*, or *C. autoethanogenum*, are used in three main application areas: for electricity generation in microbial fuel cells, for the production of methane or hydrogen in microbial electrolysis cells, and for the production of higher-value products such as butanol in microbial electrosynthesis (Patil et al. 2015; Khosravanipour Mostafazadeh et al. 2016; Rousseau et al. 2020; Raganati et al. 2022; Martínez-Ruano et al. 2024). Several application attempts in microbial electrosynthesis can be found in the literature in connection with *C. acetobutylicum*, whereby these can be defined in more detail as electro-fermentation (Guerrero et al. 2022; Hengsbach et al. 2023; Nailwal et al. 2024). The term microbial electrosynthesis covers all microbially catalyzed electrochemical processes in which CO_2_ is reduced using electrons as the driving force. In contrast, the term electro-fermentation refers to fermentation processes on organic carbon compounds in which electrons influence the metabolic pathway by changing the intracellular redox potential. Here, the electrons do not serve as the driving force of fermentation, but act as a trigger that influences the metabolism (Moscoviz et al. 2016). This technique was already used by Peguin et al. (1994) to increase the butanol yield in a batch cultivation of *C. acetobutylicum* using the mediator methyl viologen and an electric potential of − 460 to − 660 mV compared to a normal hydrogen electrode (Table 2).

**Table 2:** Overview of selected electro-fermentation attempts of *C. acetobutylicum*

Parameter	Peguin et al. (1994)	Engel et al. (2019b)	Guerrero et al. (2022)	Hengsbach et al. (2023)	Nailwal et al. (2024)
Butanol titer (g·L^−1^)	6.9	6.7	8.38	9.22	6.67
Y_Butanol/C-source_ (g·g^−1^)	0.42	0.135 ± 0.005	0.429 ± 0.004	0.207 ± 0.019	N/A
Average increase BtOH (yield or titer) (%)	51 (yield)	31.07 (yield)	33.87^a^ (titer)	20.14 ± 3.66 (yield)	68.86 (titer)
Total solvents (ABE) (g·g^−1^)	0.48	0.141 ± 0.022	N/A	0.348 ± 0.045	N/A
Carbon source (g·L^−1^)	Glucose, 66	Glucose, 45	Glucose, 18	Glucose, 45	Glucose, 20–60
WE material	Carbon rod	Carbon fabric	Graphite rod	Carbon fiber brush	Graphite
WE surface (cm^2^)	40	8.75	N/A	90	N/A
Reference electrode	NHE	Ag/AgCl (saturated. KCl)	Ag/AgCl (3.0 M)	Ag/AgCl (saturated. KCl)	Ag/AgCl (3.5 M)
Potential (mV)	− 460 to − 660	− 600	− 850	− 800	− 400
Mediator	Methyl viologen	w/o	Neutral red	w/o	w/o
Mode/type	Batch/two-chamber BES	Batch/two-chamber BES	Batch/two-chamber BES	Batch/one-chamber BES	Batch/one-chamber BES
Special aspects			Supplemented with butyrate		

^a^Calculated according to values from the corresponding reference

*ABE* acetone-butanol-ethanol, *BES* bioelectrochemical system, *NHE* normal hydrogen electrode, *N/A* not available, *w/o* without, *WE* working electrode, *Y* yield

This observation has been further investigated in recent years. Not only has the increase in yield been demonstrated with the use of methyl viologen, but also with neutral red or even without the addition of an external electron mediator (Engel et al. 2019b; Guerrero et al. 2022). For instance, Engel et al. (2019a) were able to show for the first time in a BES that an applied potential of − 600 mV against Ag/AgCl (saturated KCl) without external mediators led to an increase in the flavin concentration, in particular the proportion of flavin adenine dinucleotide, in the supernatant. Since flavins can act as electron mediators, these results suggest that *C. acetobutylicum* is able to secrete flavins to accept electrons from the electrode via flavin-based extracellular electron transfer. Furthermore, a direct electron transfer in bioelectrochemical systems has been described for *C. acetobutylicum*, which can lead to higher butanol yields. In a bench-top BES with a potential of − 800 mV, a butanol yield increase of up to 20.14% was reported (Hengsbach et al. 2023). In a smaller H-cell scale, this improvement could even be increased to 31.07% (Engel et al. 2019b). Due to these remarkable effects on solvent production, Engel et al. (2020) further investigated the mechanisms of direct electron transfer. By comparing the formation of cell appendages between biofilms in a BES and conventional biofilms, they were able to show that the cells in the BES have a higher number and density of filamentous appendages that are in direct contact with the electrode surface. Additionally, the conductivity of these filamentous structures was demonstrated using conductive atomic force microscopy, which supports the assumption that *C. acetobutylicum* is able to interact with an electrode via direct electron transfer. All these results indicate the electroactivity of *C. acetobutylicum*. But what specific effects do the uptaken electrons have on the metabolism? Various studies show that the applied electric potential influences the biphasic metabolism, which manifests itself in an earlier transition from acidogenesis to solventogenesis (Engel et al. 2019b; Nailwal et al. 2024). Moreover, Nailwal et al. (2024) were able to demonstrate an upregulation of central metabolic enzymes in a BES compared to the control. These enzymes include, among others, 3-hydroxybutyryl-CoA dehydrogenase, enoyl-CoA hydratase, and butanol dehydrogenase, all of which play a crucial role in butanol production.

Although the fermentation of *C. acetobutylicum* in BES offers a promising opportunity to enhance solvent production, the currently improved yields in the specialized reactors have not yet reached the levels of optimized conventional fermentations. Among other things, this discrepancy is attributable to challenges in scaling up the systems and optimizing the medium respectively electrolyte, as well as refining the production strain.

### Metabolic engineering of *Clostridium acetobutylicum*

Metabolic engineering remains a key approach to increasing butanol yields of *C. acetobutylicum* by improving its solvent tolerance. These methods enable selective improvements of the production strain, which leads to a significant increase in the efficiency and stability of the fermentation processes and can considerably optimize the industrial application of the microorganism.

A major limiting factor is the toxicity of organic solvents, especially n-butanol, which has an inhibitory effect on the production strain. Increasing the solvent tolerance of *C. acetobutylicum* is therefore a central goal of many research approaches (Gao et al. 2020, 2021). For decades, work has been carried out to reduce toxicity to the organism and increase butanol production, primarily by developing more robust strains using metabolic engineering and mutagenesis. Since the establishment of one of the first stable transformation protocols for *C. acetobutylicum*, numerous other genetic manipulation strategies have been developed (Mermelstein and Papoutsakis 1993). These include the targeted downregulation of genes, the overexpression of genes on plasmids, and the inactivation of specific genes in knockout mutants by homologous recombination or intron-based gene defects (Mermelstein et al. 1992; Green et al. 1996; Desai and Papoutsakis 1999; Dai et al. 2021; Ehsaan et al. 2024). In recent years, the arsenal of these strategies has been expanded by the introduction of CRISPR/Cas9-based genome editing, which enables precise, targeted, and more efficient genetic modifications of *C. acetobutylicum*. The potential of these developments is to further optimize solvent production and significantly improve the industrial use of the organism (Li et al. 2016). As a result, the cas9 gene of *Streptococcus pyogenes* has already been successfully integrated into the genome of *C. acetobutylicum*. The integration was performed under the control of a xylose-inducible system from *Clostridium difficile*, without negatively affecting the growth or solvent production of the organism. The strategy allowed high editing efficiency on multiple target genes, further highlighting the potential of the system for precise genetic modification (Wilding-Steele et al. 2021). Using CRISPR/Cas9, the influence of the inactivation of the three genes *bdhA*, *bdhB*, and *bdhC* coding for a butanol dehydrogenase in *C. acetobutylicum* was investigated. The results revealed that the inactivation of these genes significantly impairs solvent production, underlining their crucial role in butanol synthesis (Wasels et al. 2020).

Alongside initial studies using CRISPR/Cas9, there are many research approaches using the aforementioned methods. Knocking out the phosphate acetyltransferase (*pta*) and butyrate kinase (*buk*) genes combined with simultaneous overexpression of an alcohol dehydrogenase resulted in an increase in butanol production by 60% to 18.9 g∙L^−1^ (Jang et al. 2012). Another strategy to increase butanol production by *C. acetobutylicum* ATCC55025 was the knockout of different histidine kinase genes related to the phosphorylation of Spo0A, a global regulator. On the one hand, a single deletion of the kinase *cac3319* led to the defect in sporulation, but also to a higher solvent tolerance. By combining this deletion with the knockout of the kinase gene *cac0323*, an increased butanol production of over 20 g·L^−1^ was observed (Du et al. 2021). By additionally eliminating acetone synthesis in the same histidine kinase mutant by knocking out the acetoacetate decarboxylase gene *adc*, the acetone titer could be reduced to 0.1 g·L^−1^. This led to an improved butanol-to-ABE ratio of up to 87.6%. By additionally overexpressing the acetaldehyde/alcohol dehydrogenase gene *adhE2*, a mutant with a butanol production of 19.7 g·L^−1^ and a productivity of 0.41 g·L^−1^·h^−1^ was generated (Du et al. 2022). The heterologous expression of genes from other organisms for optimization is another approach to increase the solvent production of *C. acetobutylicum*. Thus, overexpression of pyruvate decarboxylase from *Zymomonas mobilis* in recombinant strains showed improved ABE production. In addition, this approach resulted in a shift of the product ratio in favor of ethanol and butanol, whereby a ratio of up to 5.26 (BE/A) could be achieved (Dharani et al. 2021). Increasing the resistance of the cell membrane to butanol can also be accomplished by targeted mutations. Mutants from recent studies generated by carbon ion beam irradiation showed significantly reduced cell surface hydrophobicity, a key factor for increased butanol tolerance. This adaptation allowed the organism to grow at butanol concentrations of up to 21.27 g·L^−1^, which was 6.04 g·L^−1^ higher than the control strain *C. acetobutylicum* ATCC824 (Gao et al. 2021).

Considerable progress has been made in recent years in overcoming key bottlenecks in the fermentation of *C. acetobutylicum*. These challenges included the inadequate genetic tools for clostridial strain development criticized by Moon et al. (2016). However, recent advances in CRISPR/Cas9 technology offer promising approaches to optimize *C. acetobutylicum* on genetic levels. In particular, the combination of modeling approaches and new genetic methods enables targeted screening of essential genes. In a recent study, 418 essential genes required for in vitro growth were identified, offering potential targets for further genetic enhancements to improve fermentation performance (Delarouzée et al. 2024).

### Co-cultivation

The limitations of *C. acetobutylicum* monoculture, such as solvent toxicity, limited substrate spectrum and oxygen tolerance, have led to the emergence of a new, innovative cultivation strategy. Inspired by natural microbial interactions, the co-cultivation of microorganisms is being widely explored for its potential to increase productivity, efficiency, and versatility in biotechnological applications (Kapoore et al. 2022). Co-cultivation strategies are also becoming more prominent in the cultivation of clostridia aiming to increase solvent production, expand the substrate spectrum to utilize low-cost feedstocks, reduce solvent toxicity, increase aerotolerance, and broaden the range of products (Cui et al. 2021; Du et al. 2020).

Recent literature highlights several co-culture approaches for *C. acetobutylicum*; each is defined by a different interaction pattern between the microorganisms (Table 3). In a commensal co-culture, one microorganism benefits by utilizing the by-products or metabolites of another, which remains unaffected. This type of interaction was observed in studies including co-cultures of different clostridia strains with *S. cerevisiae.* It was found that amino acids, secreted by yeast in response to stress conditions, positively influence solvent production by clostridia (Luo et al. 2015; Wu et al. 2019). Compared to monoculture, co-culturing *C. acetobutylicum* resulted in increased concentrations of butanol and ABE using different feedstocks. However, butanol concentration could only be increased by a maximum of 8% (Table 3). A maximum increase in ABE concentration of 27.5% was achieved in a co-culture approach by Capilla et al., primarily due to the contribution of ethanol production from yeast (Capilla et al. 2022). But still, this co-culture approach remains limited by butanol toxicity, although the additional ethanol production by the yeast resulted in increases in total ABE concentration. To maximize substrate versatility, different clostridia species with complementary substrate utilization capabilities can be integrated to form a division of labor co-culture, where each species functions independently to process specific substrates. *Clostridium* *pasteurianum* is known for its ability to use glycerol as the sole carbon source, a capability that *C. acetobutylicum* does not share (Biebl 2001). Regarding the utilization of renewable feedstocks, employing a combination of these clostridia species could be advantageous for utilizing different sugar types. In a fermentation based on food waste, the butanol titer was increased from 8.2 g·L^−1^ in monoculture of *C. acetobutylicum* to 13.2 g·L^−1^ in co-culture (Zhang et al. 2021). Experiments based on glucose also revealed that the solvent concentration in the co-culture was nearly double that of a monoculture of *C. acetobutylicum*. Notably, *C. pasteurianum* typically achieves higher solvent concentrations through the production of 1,3-propanediol. However, butanol concentration also increased by up to 40%, although the specific reasons for this increase remain unclear (Zhang et al. 2021). Equally promising for the utilization of renewable feedstocks in solvent production is the integration of bacteria or fungi with cellulolytic activity, forming a syntrophic co-culture where cellulolytic organisms break down cellulose into fermentable sugars, which are then utilized by solvent-producing *C. acetobutylicum*. For instance, utilizing municipal solid waste as a substrate, the solvent concentration can be increased from 11.7 to 17.1 g·L^−1^ by combining *C. acetobutylicum* with the facultative anaerobic bacterium *Enterobacter aerogenes* (Ebrahimian et al. 2022). To achieve these solvent concentrations, however, a pretreatment is still required, involving ethanolic organosolv treatment followed by enzymatic hydrolysis of cellulose and hemicellulose (Ebrahimian et al. 2022). Co-culture strategies also offer opportunities to broaden the product spectrum, particularly in the production of esters, which are valuable both as solvents and in biofuel applications. To facilitate ester production through lipase-mediated esterification, pairing *C. acetobutylicum* with an acid-producing microorganism can effectively yield the essential precursors of esters. For example, butanol and butyric acid can be produced through the combination of *C. acetobutylicum* and *Clostridium tyrobutyricum*, leading to the formation of the ester butyl-butyrate (Lu et al. 2023). Similarly, *C. acetobutylicum* can be combined with *Actinobacillus succinogenes* to produce the ester butyl-acetate (Lv et al. 2021). In this way, the production of esters derived from renewable raw materials is enabled. Overall, utilizing co-cultures with *C. acetobutylicum* is an innovative strategy for generating not only butanol but also other valuable products from diverse renewable feedstocks.

**Table 3:** Overview of co-cultivation approaches including *C. acetobutylicum*

Strains	Substrate	Cultivation methods	*C. acetobutylicum* monoculture, product, g·L^−1^	co-culture, product, g·L^−1^	Intention	Reference
*C. acetobutylicum*, *S. cerevisiae*	Rice straw hydrolysate	pH > 4.8, 37 °C, 120 rpm, inoculation with 5% v/v each strain, addition of *S. cerevisiae* after 5 h	Butanol: 6.5 ABE: 9.5	Butanol: 7.0 ABE: 13.1	Improve xylose uptake by amino acid secretion from yeast	Capilla et al. (2022)
*C. acetobutylicum*, *S. cerevisiae*	Corn flour hydrolysate	pH 5, 37 °C, inoculation with *C. acetobutylicum* 10% v/v, addition of *S. cerevisiae* after 24 h with 0.2 g-DCW/L	Butanol: 12.87 ABE: 21.79	Butanol: 13.95 ABE: 24.23	Enhance butanol synthesis by amino acid secretion from yeast, enhance substrate uptake by competition with yeast	Luo et al. (2015)
*C. acetobutylicum*, *S. cerevisiae*	Cassava	37 °C, inoculation with 5% v/v each strain	Butanol: 11.8 ABE: 19.0	Butanol: 11.4 ABE: 21.7	Enhance solvent production by co-culturing amylolytic clostridia strain with non-amylolytic yeast	Qi et al. (2018)
*C. acetobutylicum*, *C. pasteurianum*	Glucose	37 °C, 150 rpm, inoculum 10% v/v, ratio *C. acetobutylicum*: *C. pasteurianum* 0.30	Butanol: 9.9 solvent: 18	Butanol: 12.1 ABE: 23.1	Enhance substrate utilization	Kumar et al. (2024)
*C. acetobutylicum, C. pasteurianum*	Glucose	37 °C, 108 rpm, Inoculum 10% v/v, ratio *C. acetobutylicum*: *C. pasteurianum* 7:3	Butanol: 8.2 solvent: 12.5	Butanol: 13.2 ABE: 23 L	Enhance solvent production	Zhang et al. (2021)
*C. acetobutylicum*, *Enterobacter aerogenes*	Municipal solid waste hydrolysate	37 °C, 150 rpm, inoculation with 6% v/v each strain	Butanol: 7.7 Solvents: 11.7	Butanol: 8.4 solvents: 17.1	Enhance solvent and hydrogen production	Ebrahimian et al. (2022)
*C. acetobutylicum*, *Enterobacter hormaechei* subsp.* xiangfangensis*	Glucose and alginate (1:1)	37 °C, Inoculum 2% v/v of seed co-culture	Butanol: 5.6 Solvents: 14.75	Butanol: 9.0 Solvents: 20.32	Use alginate as substrate	Dharshini et al. (2022)
*C. acetobutylicum*, *Nesterenkonia* sp.	Untreated potato starch	35 °C, inoculation with 1.2 mg CDW/L *Nesterenkonia* and 15 mg CDW/L *C. acetobutylicum*, inoculation *C. acetobutylicum* after 1.5 h	Butanol: 5.4 ABE: 7.2	Butanol: 7.0 ABE: 9.3	Utilize starch as substrate by co-culturing amylase-producing bacterium	Ebrahimi et al. (2020)
*C. acetobutylicum*, *Thermoanaerobacterium thermosaccharolyticum*	Xylan	Start with inoculation of *T. thermosaccharolyticum,* 55 °C for 50 h, inoculation with *C. acetobutylicum* at 50 h, temperature adaption to 37 °C, strain ratio: 1:1	N/A	Butanol: 13.3 ABE: 21.9	Degradation of hemicellulose by co-culturing with cellulolytic bacteria	Jiang et al. (2020)
*C. acetobutylicum*, *Neocallimastix californiae*	Reed canary grass	39 °C, Start with inoculation of *N. californiae*, inoculation with *C. acetobutylicum* after 22 days	N/A	Butanol: 0.7	Degradation of lignocellulose by co-culturing with cellulolytic fungi	(Brown et al. 2023)
*C. acetobutylicum*, *C. tyrobutyricum*	glucose	pH 5, 37 °C, 180 rpm, Inoculation ratio (OD600) *C. acetobutylicum*: *C. tyrobutyricum* 3:2, 4% v/v, Addition of *E. coli* strain with surface displayed lipase, ester extraction with dodecane	N/A	Butyl-butyrate: 6.7	Facilitate ester production	Lu et al. (2023)
*C. acetobutylicum*, *A. succinogenes*	glucose	37 °C, pH 5.5, start with inoculation of *C. acetobutylicum*, inoculation with *A. succinogenes* after 96 h, inoculation ratio 1:5, addition of lipase, ester extraction with dodecane	N/A	Butyl-acetate: 2.2	Facilitate ester production	Lv et al. (2021)

*N/A* not available

## Conclusion and outlook

The ABE fermentation with *Clostridium acetobutylicum* has a long history spanning over 100 years, characterized by significant technological advancements and challenges. Originally popular in the industrial production of butanol, the method experienced a decline due to the availability of inexpensive fossil fuels and technical difficulties. However, in recent years, the growing interest in sustainable and renewable energy has led to a revival of this technology. Challenges in the cultivation process of *C. acetobutylicum* such as strain degeneration, acid crash, solvent toxicity, low product yields, and high cost of substrate are currently being re-examined to find solutions based on recent research. For research to progress, it is particularly important to present a well-documented cultivation process, which serves as the basis for comparable and reproducible results. For solventogenic clostridia, it is particularly important to highlight the pre-culture, as its cultivation affects acid and solvent production in the main culture. Documentation of pre-culture methods should therefore be comprehensive including information on inoculum, media, pH, and metabolic phase at the time of cell harvest. In the cultivation of *C. acetobutylicum* in batch but also in continuous culture, pH is an important parameter to influence the metabolic shift from acidogenesis to solventogenesis. In continuous fermentation, the use of immobilized cells leads to further improvements in the productivity and stability of the process. The implementation of in situ product removal techniques, including gas stripping, liquid–liquid extraction, and adsorption, provides solutions to overcome the product toxicity. Additionally, advancements in solvent tolerance of *C. acetobutylicum* achieved through metabolic engineering reveal promising potential for further optimization. This offers key solutions to address the primary limitation of solvent toxicity and could lead to a further breakdown of the metabolic properties of ABE fermentation with *C. acetobutylicum*. Moreover, higher product concentrations also simplify the purification process. Other innovative process strategies include utilizing renewable raw materials, such as lignocellulosic biomass. This approach not only enhances sustainability by transforming side streams from agriculture and food production into valuable biobutanol but also reduces reliance on fossil resources. Further promising research areas focus on co-cultivation with other beneficial microbes and the application of bioelectrochemical systems. The combination of these research areas offers the potential for considerable progress.

Advances in cultivation strategies, metabolic optimization, and substrate selection have reawakened the potential of ABE fermentation. Thus, *C. acetobutylicum* is regaining attention as a promising candidate for sustainable butanol production. The growing scientific interest in *C. acetobutylicum* in recent years highlights the successful research efforts in this field. Given the promising potential of the process, this trend should continue in order to re-establish its potential for industrial application and take a further step towards a bio-based economy.
